# Allergic sensitization to peanuts is enhanced in mice fed a high-fat diet

**DOI:** 10.3934/allergy.2020008

**Published:** 2020-10-10

**Authors:** Joseph J. Dolence, Hirohito Kita

**Affiliations:** 1 Department of Biology, University of Nebraska at Kearney, Kearney, NE 68849; 2 Department of Medicine and Immunology, Mayo Clinic Scottsdale, Scottsdale, AZ 85259

**Keywords:** peanut allergy, obesity, high fat diet, allergic disease, anaphylaxis, Western diet

## Abstract

The incidence of peanut (PN) allergy is on the rise. As peanut allergy rates have continued to climb over the past few decades, obesity rates have increased to record highs, suggesting a link between obesity and the development of peanut allergy. While progress has been made, much remains to be learned about the mechanisms driving the development of allergic immune responses to peanut. Remaining unclear is whether consuming a Western diet, a diet characterized by overeating foods rich in saturated fat, salt, and refined sugars, supports the development of PN allergy. To address this, we fed mice a high fat diet to induce obesity. Once diet-induced obesity was established, mice were exposed to PN flour via the airways using our 4-week inhalation model. Mice were subsequently challenged with PN extract to induce anaphylaxis. Mice fed a high-fat diet developed significantly higher titers of PN-specific IgE, as well as stronger anaphylactic responses, when compared to their low-fat diet fed counterparts. These results suggest that obesity linked to eating a high-fat diet promotes the development of allergic immune responses to PN in mice. Such knowledge is critical to advance our growing understanding of the immunology of PN allergy.

## 1. Introduction

The rate of obesity has risen substantially in the last three decades, culminating in approximately 40% of adults and 19% of children in the United States being obese in a recent study [1]. The prevalence of peanut (PN) allergy is also increasing rapidly [2]. The rate in which this disease has expanded over the past two decades outpaces what can be explained solely by genetics, suggesting a strong role for environmental factors. The Western diet, a diet characterized by an overindulgence of low-fiber foods rich in saturated fat, salt, refined sugars, coupled with reduced consumption of nutrient-rich foods, such as fruits and vegetables, has been implicated as a reason for the increased incidence of allergies [3-5]. Eating a Western diet has been shown to impact the microbial communities in our guts, leading to a decrease in microbial diversity and disruption in normal host-microbe interactions [6-14]. Furthermore, studies using mouse models have shown that the gut microbiomes function to induce tolerance to food allergens, including PN, and alterations in the microbial flora lead to allergic sensitization [15,16]. Collectively, these findings suggest a connection between consuming a Western diet and increasing prevalence of food allergies. In support, a recent study showed that feeding mice a high-fat diet promoted the development of allergy to a model antigen ovalbumin (OVA) [5]. Still unclear, however, is the impact diet has on PN allergy. Therefore, the goal of this study was to examine whether eating a high-fat diet promotes the development of PN allergy.

To accomplish this, we fed mice either a chow containing 45% fat (high-fat diet or HFD) or a control chow with 10% fat (low-fat diet or LFD). Once obesity was established, both cohorts of mice were exposed to PN using our established inhalation model [17]. To quantify allergic sensitization to PN, serum was tested for the presence of PN-specific antibodies and mice were challenged with PN to induce anaphylaxis. Using these approaches, we identified that mice fed a high fat diet developed higher serum levels of PN-specific IgE and stronger anaphylactic reactions. These data suggest that eating a HFD enhances the ability of immune cells to mount an allergic response to PN.

## 2. Materials and methods

### 2.1. Mice

Three-week old female BALB/c mice were obtained from The Jackson Laboratory (Bar Harbor, ME) and placed on either a high-fat diet (HFD) to induce obesity or a control low-fat diet (LFD) (described in more detail below—see section 2.2). Male mice were not used in these experiments. Mice were housed in standard pathogen-free conditions under ad libitum feeding conditions. Mice exposed to PBS or PN were marked individually (e.g. LFD PBS-1, LFD PBS-2, LFD PN-1, HFD PBS-1, HFD PN-1, etc.) randomly on the day of retroorbitally bleeding (day 27) with a marker on their tails, and these identification markings allowed for the monitoring of rectal temperature and tracking mouse behavior following challenge with PN the next day (day 28).

### 2.2. Induction of diet-induced obese mouse model

Mice were split into two cohorts and either fed a HFD (45% energy by fat) or a standard LFD (10% energy by fat) for at least 29 weeks to establish diet-induced obese BALB/c mice (see Figure 2a for experimental schematic). Diets were purchased from Research Diets, Inc. (New Brunswick, NJ) and specifics about the diets can be found in Table 1. Mice were weighed weekly to track that mice fed HFD displayed significant increase in body weight, and blood was collected retroorbitally at 8- and 15-week intervals to test plasma leptin levels with ELISA (described below). Weight and leptin levels were metrics used to document that mice fed HFD have established obesity compared to mice fed standard LFD. Following induction of diet-induced obesity, mice were exposed to PN by inhalation as described below—see section 2.4.

### 2.3. Allergens

Peanut (PN) flour was purchased from the Golden Peanut Company (Alpharetta, GA). We tested endotoxin levels in the flour by Limulus Amebocyte Lysate assay (Lonza, Walkersville, MD) and found undetectable levels (<0.5 EU/mg flour). Crude PN extract was purchased from Greer Laboratories (Lenoir, NC) for intraperitoneal challenge.

### 2.4. Model to induce peanut allergy via inhalation

Naïve BALB/c mice fed either HFD to induce obesity or LFD as control were sensitized with PN flour as we have described previously [17]. In each experiment, mice were split into four groups: LFD PBS, LFD PN, HFD PBS, and HFD PN. In total across two experiments, 5 mice were in each PBS group, 6 mice were in the LFD PN group, and 8 mice were in the HFD PN group. Briefly, mice were exposed to either 100 μg peanut flour in 50 μL PBS or PBS alone twice per week for 4 weeks. On day 27, mice were retroorbitally bled to determine serum levels of PN-specific IgE and IgG1. The next day (day 28), mice were challenged with 2.5 mg PN peanut extract in 500 μL PBS via intraperitoneal injection to induce anaphylactic reaction. Rectal temperature and clinical symptoms were monitored before (0 min) and after PN challenge (every 10 min for 1 h). Rectal temperatures were recorded with an electronic thermometer (Oakton Instruments, Vernon Hills, Ill) equipped with a RET-3 rectal probe (Physitemp Instruments, Clifton, NJ). Clinical symptoms were scored based on the following published criteria [18]: 0, no symptoms; 1, scratching of ear and mouth; 2, puffiness around eyes and mouth, pilar erection, labored breathing; 3, prolonged period of motionlessness; 4, severely reduced motility, tremors, severe respiratory distress; and 5, death.

### 2.5. ELISA

Serum levels of PN-specific IgE and IgG1 were measured by ELISA as previously described [17]. Leptin was measured in plasma samples using a commercial mouse leptin sandwich ELISA kit (R&D Systems, Minneapolis, MN) according to manufacturer instructions.

### 2.6. Ethics approval of research

All animal experimental protocols and procedures were carried out with the approval of the Mayo Clinic Institutional Animal Care and Use Committee under IACUC protocol number A38914.

### 2.7. Statistics

Differences between the various treatment groups were deemed statistically significant using a Student *t* test, with p ≤ 0.05 considered statistically significant. Numerical data are presented as mean ± SEM. Error bars within figures represent SEM.

## 3. Results

### 3.1. Establishment of diet-induced obese mouse model

**Figure 1.**
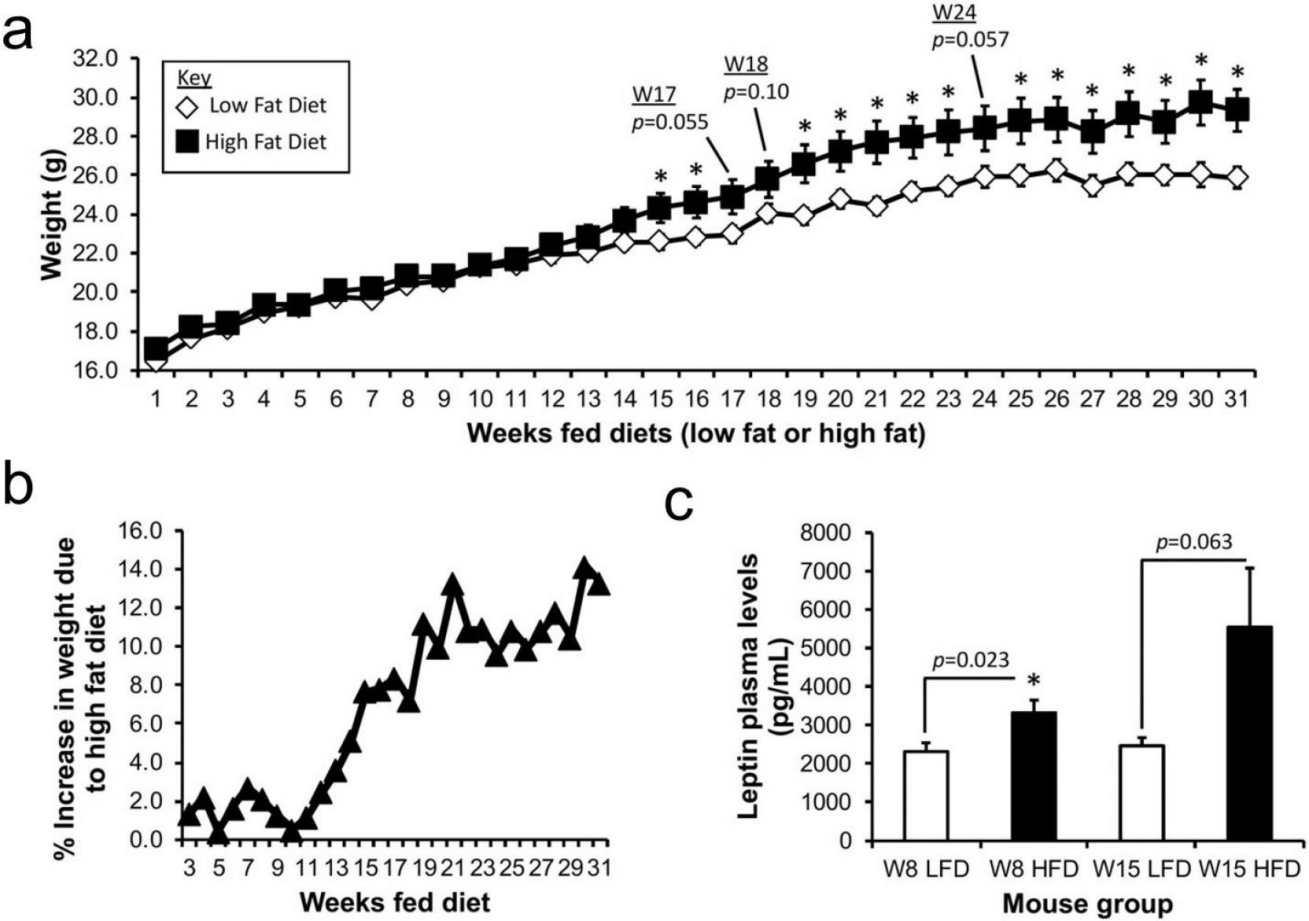
Establishment of a mouse model of diet-induced obesity. (a) Mice were fed with either high fat (45% energy by fat) or a standard, low fat (10% energy by fat) diet for at least 29 weeks to establish obese or control mice prior to sensitization to peanut (PN) via inhalation. Mice were weighed weekly and their weights are reported in grams. Data represents a mean ± SEM of 24 mice per a diet group. (b) Percent increase in weight of mice fed HFD as compared to mice fed LFD. Data are shown starting 3 weeks after diet commenced and is recorded weekly through week 31. (c) Leptin plasma levels were measured after 8 and 15 weeks via ELISA. Data is the mean ± SEM of 9 of the 24 mice per diet group randomly selected to test for leptin levels in plasma. For both (a) and (c), * *p* < 0.05 compared to control mice fed LFD, unless indicated otherwise.

The goal of this study was to investigate whether obesity increases the susceptibility of developing PN allergy. Beginning at 3 weeks of age, BALB/c female mice were fed either a HFD to induce obesity or a control and standard LFD for at least 29 weeks prior to PN exposure. After 16 weeks of feeding a HFD, mice typically exhibit a 20–30% increase in body weight, along with other signs of obesity (e.g. adipocyte hyperplasia, mesenteric fat deposition, increased fat mass, diabetes, and hypertension) compared to mice fed control chow [19,20]. Similar to these published findings, starting after 15 weeks of eating the HFD chow, mice in our study displayed a significant increase in weight when compared to their LFD chow fed counterparts (Week 15 HFD: 24.32 ± 0.75 g; Week 15 LFD: 22.58 ± 0.39, *p* = 0.046 (Figure 1a). Mice fed HFD exhibited 7.82% weight gain by week 16, and from weeks 19–31, consistently showed a 10–13% higher body weight as compared to LFD-fed mice (Figure 1b). These findings are consistent with BALB/c mice being more resistant to HFD induced obesity than other mouse strains [19]. To further verify our model, leptin levels, which are known to be elevated in obese mice fed HFD [21,22], were measured in plasma at 8 and 15 weeks after feeding commenced (Figure 1c). As obesity develops, plasma leptin levels are known to increase [23]. Mice fed HFD exhibited higher leptin levels at both time points (Week 8 LFD: 2316 ± 232 pg/mL; Week 8 HFD: 3324 ± 329 pg/mL, *p* = 0.023; Week 15 LFD: 2468 ± 213 pg/mL; Week 15 HFD: 5549 ± 1527 pg/mL, *p* = 0.063). Collectively, these data strongly support that we developed diet-induced obese and appropriate control mice necessary to examine how diet impacts sensitization to PN.

### 3.2. HFD mice develop higher PN-specific IgE responses

**Figure 2.**
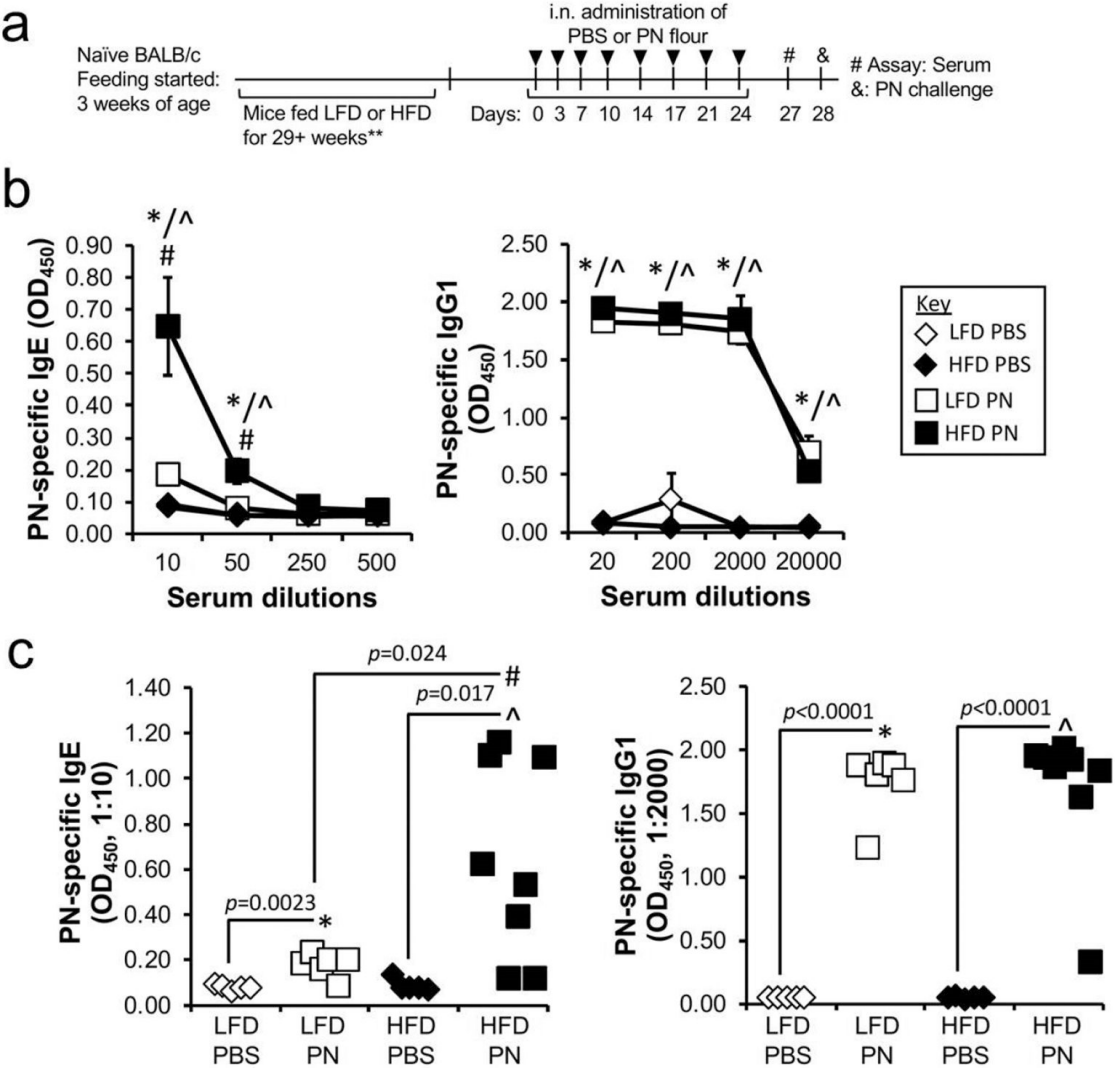
Mice fed HFD produce significantly higher PN-specific IgE than those fed LFD. (a) Schematic showing timeline of experiment. Mice were fed starting at 3 weeks of age and fed for 29+ weeks (** one group of mice was fed for 29 weeks and the other group of mice were fed for 56 weeks, so the mice were 8–14 months of age at the time of sensitization by exposure to PN flour). (b) Levels of PN-specific antibodies in d27 sera were measured by ELISA. Data are pooled from 2 experiments and represented as a mean ± SEM (n = 5 in each PBS group, 6–8 mice in each PN group). (c) Scatter plots showing PN-specific antibody levels of each mouse are shown. For both (b) and (c), * reveals significance (*p* < 0.05) between LFD PBS and LFD PN groups, ^ indicates significance (*p* < 0.05) between HFD PBS and HFD PN, and # depicts significance (*p* < 0.05) between LFD PN and HFD PN.

HFD and LFD control mice were sensitized to PN using our 4-week inhalation model (Figure 2a) [17]. Briefly, mice were exposed to PN flour (or PBS vehicle) twice per week for 4 weeks. On day 27, blood was collected to analyze the presence of PN-specific antibodies. Regardless of diet, mice exposed to PN produced PN-specific IgE and IgG1 responses (Figure 2b,c). Strikingly, HFD mice developed 3.5-fold higher titers of PN-specific IgE following PN inhalation than their LFD PN counterparts (OD_450_ HFD PN: 0.648 ± 0.153; OD_450_ LFD PN: 0.183 ± 0.021 , *p* = 0.024). In contrast, such differences were not observed in the development of PN-specific IgG1 (Figure 2b,c).

### 3.3. HFD mice sensitized to PN undergo more severe systemic anaphylactic reactions following PN challenge

**Figure 3.**
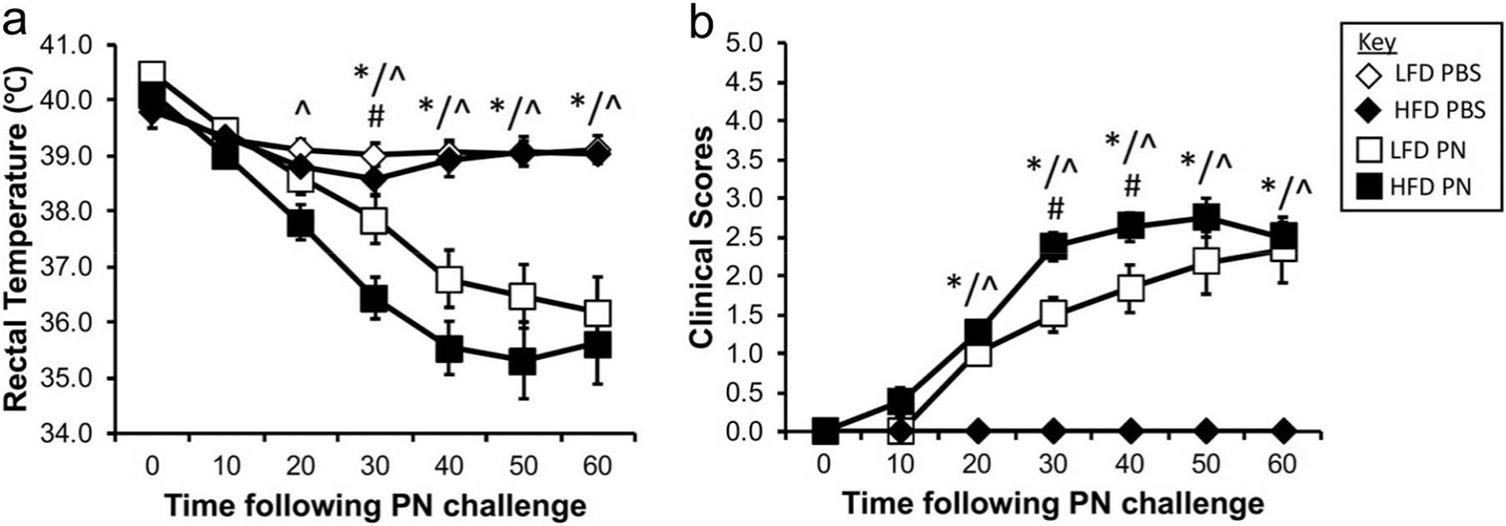
Mice fed HFD undergo more severe anaphylactic reactions upon PN challenge than LFD-fed counterparts. To track anaphylaxis to PN in mice, rectal temperature (a) and clinical scores (b) were recorded for 60 min following intraperitoneal injection with peanut extract on day 28. Additional description of clinical score values can be found in the materials and methods section. Data are pooled from 2 experiments and represent mean ± SEM (n = 5 in each PBS group, 6–8 mice in each PN group). * reveals significance (*p* < 0.05) between LFD PBS and LFD PN groups, ^ indicates significance (*p* < 0.05) between HFD PBS and HFD PN, and # depicts significance (*p* < 0.05) between LFD PN and HFD PN.

On day 28, mice were challenged intraperitoneally with PN extract to elicit an anaphylactic reaction. To track the progress of anaphylaxis, rectal temperatures and clinical scores were monitored every 10 min for 1 h following PN challenge. Both LFD and HFD mice sensitized to PN underwent anaphylaxis (Figure 3). Not surprisingly, PBS-sensitized mice did not react to PN challenge. Interestingly, HFD mice underwent a more severe anaphylactic reaction when compared to LFD control mice. Thirty minutes post-challenge, PN-sensitized HFD mice displayed a larger drop in rectal temperature (HFD PN: 36.4 ± 0.4 °C; LFD PN: 37.9 ± 0.4 °C, *p* = 0.029) and higher clinical score (HFD PN: 2.4 ± 0.2; LFD PN: 1.5 ± 0.2, *p* = 0.01) compared to LFD control mice sensitized with PN (Figure 3). Clinical scores of PN-exposed HFD mice remained significantly higher than LFD PN at the 40-minute time point. Overall, these data suggest that HFD mice sensitized to PN develop the symptoms of systemic anaphylaxis (i.e. drop in body temperature and presence of clinical signs) faster and more severely than LFD PN mice following challenge with PN.

## 4. Discussion

A recent study has suggested that feeding mice a HFD promotes allergic sensitization to a model antigen OVA likely through diet-induced changes to the gut microflora [5]. Since alterations in the gut microbiome has been linked to sensitization to PN [16], we wondered what impact diet had on the development of PN allergy. To address this question, we used a straightforward approach to first develop diet-induced obese mice, along with control chow fed mice, and second, expose these mice to PN via our inhalation model [17]. We found that mice that consumed HFD developed higher titers of PN-specific IgE. The anaphylactic reactions following PN challenge were more severe than their control chow fed counterparts. These data suggest that eating HFD creates an inflammatory environment that promotes developing allergic immune responses to PN. Furthermore, these data provide additional evidence that supports the notion that eating HFD, the diet commonly associated with a Western lifestyle, is one of the reasons for an increased prevalence of allergies [3-5].

It has been reported previously that the gut microbiota changes after consuming HFD [6-14]. Microbial diversity declines and host-microbe interactions are altered. It is also known that macrophages are activated by HFD. In obese mice, macrophages accumulate in adipose tissue where they participate in driving inflammation through the release of pro-inflammatory cytokines [24,25]. Obesity also induced mast cells and eosinophils to accumulate in the trachea and lung in an eosinophilic esophagitis (EoE) mouse model [26]. Therefore, eating a high fat diet itself may induce these cellular changes in the airways, resulting in development of EoE [26]. In another study, HFD-fed female mice presented higher numbers of leukocytes in the lung tissue, attributed to higher numbers of neutrophils, macrophages, and eosinophils verses standard chow female mice [27]. While much remains to be learned about the consequences of obesity-linked cellular accumulation in the airways, these data would suggest that the increase in adipose tissue leads to an increase in inflammatory cells in the airway capable of responding to airway allergens. In agreement, genetically obese (*ob/ob, db/db*, and *Cpe^fat^*) mice developed greater allergic responses due to inhalation of ozone, a common environmental pollutant and asthma inducer, than lean mice [28]. The susceptibility of genetically obese mice to developing PN allergy has been unknown.

While mice fed HFD displayed significantly higher levels of PN-specific IgE, no difference was observed in IgG1 titers. Intraperitoneal challenge may result in IgG1-mediated anaphylactic response via FcγRIII-expressing cells [29]. This is especially observed in IgE-deficient mice [29]. Although LFD PN mice in this study developed a significant PN-specific IgE response, the magnitude of responses was lower than we previously observed using the inhalation model [17]. Therefore, it remains possible that we failed to observe striking difference in the anaphylactic responses between HFD PN and LFD PN mice following PN challenge because IgG1 may also induce anaphylactic response in LFD PN mice. Due to lower IgE, the IgG1-mediated reaction could have been more fully manifested following challenge with PN in LFD PN mice. This finding would be consistent with data that shows both IgG1 and IgE have to be absent to fully abrogate peanut-induced anaphylaxis [29]. Future studies should examine whether in the absence of IgG1 antibody responses, more pronounced differences can be observed between LFD and HFD mice.

The specific mechanism for how HFD stimulates a more robust immune response to PN than LFD remains unclear. Recently, we have shown that a type 2 cytokine IL-13, which is secreted rapidly by group 2 innate lymphoid cells (ILC2s) following exposure to PN, is critical to drive the development of T follicular helper (Tfh) cells and production of PN-specific IgE [30]. In the same study, we also showed that IL-1α alone could induce IL-13 production from ILC2s and that the release of IL-13 caused by exposure to PN was dependent on IL-1R1 (the receptor for IL-1α and IL-1β). Furthermore, we have shown that signaling through IL-1R1 is necessary for sensitization to PN [17]. Therefore, future studies should examine whether HFD increases the presence of cells capable of secreting IL-1α into the environment to promote IL-13 production by ILC2s. Indeed, alveolar macrophages are a potential cellular source of IL-1α as they have been shown to generate IL-1α in response to inhaled fine particles [31]. Notably, mice fed HFD displayed significant increases in alveolar macrophages [32]. In agreement with the findings we describe herein, the study also showed that mice fed HFD exhibited a greater allergic response to house dust mite than control chow fed mice [32]. Taken together, it is reasonable to speculate that HFD affects one or more of the members of the immune pathway, such as alveolar macrophages, IL-1α, ILC2s, IL-13, or Tfh cells, that lead to allergic sensitization to PN via inhalation. Future studies will need to pinpoint which members of the pathway are modulated by HFD.

While obesity is well known to be linked to an increased prevalence of cardiovascular disease and type 2 diabetes, its impact on lung diseases is also well documented. Obesity is a well-known risk factor for asthma, obstructive sleep apnea, obesity hypoventilation syndrome, pulmonary hypertension, as well as affecting outcomes in acute respiratory distress syndrome and chronic obstructive pulmonary disease [33]. Obesity leads to a constant state of low-grade inflammation in pro-inflammatory macrophages that have been shown to reach up to 50% of the cellularity of subcutaneous adipose tissue in obese individuals [24,34]. In addition, obese humans and mice have been shown to display increases in mast cells in their adipose tissue [35]. Given their role as a key player in allergic responses, these data suggest that mast cells may also play a critical role in establishing the immune environment necessary to make obese individuals more susceptible to airway diseases.

We have shown previously that PN exposure through the airways elicited PN sensitization in mice, which develop systemic anaphylaxis upon PN challenge [17]. Moreover, the majority of PN-allergic children experience their first allergic reaction to PN upon first ingestion of PN [36]. The Learning Early About Peanut Allergy (LEAP) study showed early introduction of PN into the diet prevented the development of clinical PN allergy among children at high risk [37], suggesting early oral exposure may induce tolerance. Strikingly, a greater percentage of children in LEAP’S PN avoidance group developed elevated titers of PN-specific IgE antibody, suggesting sensitization to non-oral environmental PN allergens [37]. PN is readily detectable in household dust and has recently been shown to promote airway sensitization to PN in mice [38-40]. Our increased knowledge about the immunological relevance of dust to PN allergy coupled with the growing belief that young children should be orally consume PN in order to drive tolerance, strongly supports further examination of immunological pathways driving inhalation-mediated PN sensitization. Our study accomplished this task by showing that obesity linked to eating a HFD made mice more susceptible to developing allergic response to PN via the airways. Future studies will elucidate the mechanism of the response itself, but this knowledge is critical to advance our growing understanding of the immunology of PN allergy.

## 5. Conclusion

This project showed that mice fed high-fat diet and were sensitized to PN allergen generated significantly more PN-specific IgE and underwent more severe anaphylaxis upon PN challenge than low-fat diet fed counterparts, suggesting eating a high-fat diet promotes an immune environment more supportive to the development of PN allergy.

## Figures and Tables

**Table 1.** Ingredient formulation of low-fat diet (LFD) and high-fat diet used in study.

Macronutrient	Ingredient	LFD (control)* (g)	HFD** (g)
Protein	Casein, lactic, 30 mesh	200.0	200.0
Protein	Cystine, L	3.0	3.0
Carbohydrate	Sucrose, fine granulated	354.0	176.8
Carbohydrate	Starch, corn	315.0	72.8
Carbohydrate	Lodex 10 (maltodextrin)	35.0	100.0
Fat	Soybean oil, USP	25.0	25.0
Fat	Lard	20.0	177.5

* LFD was purchased for study from Research Diets, Inc. (D12450B). Caloric information broken down by % Kcal for LFD: protein—20% Kcal, fat—10% Kcal, Carbohydrate—70% Kcal. The energy density for LFD: 3.82 Kcal/g.

** HFD was purchased for study from Research Diets, Inc. (D12451). Caloric information broken down by % Kcal for HFD: protein—20% Kcal, fat—45% Kcal, Carbohydrate—35% Kcal. The energy density for HFD: 4.7 Kcal/g.
